# A X-Band Integrated Passive Device Structure Based on TMV-Embedded FOWLP

**DOI:** 10.3390/mi16060719

**Published:** 2025-06-17

**Authors:** Jiajie Yang, Lixin Xu, Xiangyu Yin, Ke Yang

**Affiliations:** 1 School of Mechatronical Engineering, Beijing Institute of Technology, Beijing 100081, China; yangjiajie@bit.edu.cn (J.Y.); 3120205167@bit.edu.cn (K.Y.); 2 School of Integrated Circuits and Electronics, Beijing Institute of Technology, Beijing 100081, China; bitxyyin@163.com

**Keywords:** integrated passive device, fan-out wafer-level package, through-mold via, antenna-in-package (AiP), ultra-thin

## Abstract

In this paper, the fabrication and testing of an integrated passive device (IPD) structure for X-band FMCW radar based on the fan-out wafer-level packaging (FOWLP) process are discussed. First, a transition line structure is added to the IPD structure to increase the upper impedance limit of the substrate, so as to reduce the process implementation difficulty and development cost. Second, the vertical soldered SubMiniature Push-On Micro (SMPM) interfaces testing method is proposed, reducing the testing difficulty of the dual-port structure with the antenna. Finally, the process fabrication as well as testing of the IPD structure are completed. The dimensions of the fabricated structure are 16.983 × 24.099 × 0.56 ${\mathrm{mm}}^{3}$. Test results show that, with a center frequency of 8.5 GHz, the actual operational bandwidth of the structure reaches 7.66% (8.095–8.74 GHz), with a maximum isolation of 33.9 dB. The bandwidth with isolation greater than 20 dB is 1.76% (8.455–8.605 GHz). The maximum gain at the center frequency is 2.02 dBi. Additionally, experimental uncertainty analysis is performed on different IPD structures, and the measurement results are basically consistent. These results validate the feasibility of the FOWLP process in the miniaturization of X-band FMCW radar antenna and other passive devices.

## 1. Introduction

X-band FMCW radar systems are widely used in applications such as weather monitoring, geological surveys, and target detection [1,2,3]. Most research focuses on miniaturizing active circuits in the RF front end [4,5,6,7,8]. However, passive components like antennas, baluns, couplers, and circulators [9] also play a crucial role in system performance. Integrating antennas and the passive components in X-band FMCW RF front ends faces several challenges, such as the mismatch between antennas and silicon-based processes [10], large passive device sizes, and high integration costs. These issues hinder the miniaturization and cost reduction of RF front-end passive structures, complicating the overall device integration.

In terms of antenna integration, Zhang et al. proposed an antenna-in-package (AiP) solution [11,12], which has been widely used in RF systems across various fabrication processes, including silicon-based [13], low temperature co-fired ceramic (LTCC) [14,15], high density interconnection (HDI) [16,17], printed circuit board (PCB) [18], and fan-out wafer-level packaging (FOWLP) [19,20,21]. Compared to other technologies, FOWLP offers a simpler process, higher interconnect density, thinner substrates, and effectively reduces parasitic parameters, package size, and manufacturing costs. Current AiP FMCW radar systems using FOWLP primarily focus on 60 GHz and 120 GHz bands, integrating the antenna horizontally with the chip. However, this method significantly increases the integration size for X-band passive devices. Furthermore, existing studies do not fully utilize the advantages of the FOWLP two-sided multilayer redistribution layer (RDL) process, which is only used to integrate the antenna without considering other microstrip passive components.

**Figure 1:**
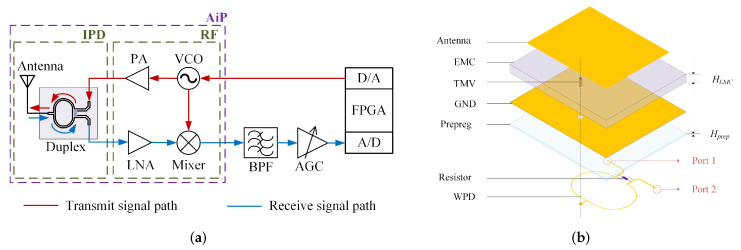
The block and structural diagrams of the IPD structure proposed in [22]: (**a**) block diagram; (**b**) exploded view of IPD based on FOWLP technology.

For the above problems, we proposed an integrated passive device (IPD) structure for X-band FMCW radar systems, as shown in Figure 1 [22]. The structure integrates the antenna and Wilkinson power divider (WPD) on the opposite sides of the substrate. The IPD structure acts as an interface between the system and the external environment, connecting the transmission link and reception link of the FMCW system. We also proposed an optimization of the IPD structure based on the FOWLP process. In the FOWLP process for fabricating the IPD structure, the through-mold via (TMV) chip is placed onto a carrier, and the epoxy molding compound (EMC) is used to encapsulate a new wafer. Then, RDL technology is used to fabricate the corresponding metal structures on both sides of the EMC, with the antenna on the top, the grounding layer, and the Wilkinson power divider used as a duplexer on the bottom. The IPD structure is vertically interconnected with the chip substrate using solder balls.

In this paper, the preparation and testing of the proposed IPD structure are accomplished using the FOWLP process. First, for the upper impedance limit problem caused by the ultra-thin single-layer substrate of the actual process, an inter-layer transition structure is proposed to enable the fabrication of the maximum impedance that can satisfy the demand of the IPD structure; after that, the fabrication process flow of the IPD structure is introduced; and then, for the testing challenge of the dual-port ultra-thin structure with antennae, a test scheme of vertically soldering the SubMiniature Push-On Micro (SMPM) interfaces is proposed, and the electromagnetic simulation is performed; finally, the prepared structure is tested to verify the potential of the IPD structure based on FOWLP process for application in an X-band FMCW system.

## 2. Practical Process-Driven Design of the Integrated Passive Device Structure

### 2.1. Revised IPD Structure for Enhanced Process Feasibility

In the literature [22], the ideal simulation model of the IPD structure is presented and the related process parameters are provided, and the thickness of the dielectric layer that is used as the substrate for the power divider is 30 µm. However, in the RDL process, thicker dielectric layers are more difficult to control during processes such as coating, lithography, and etching, increasing the process complexity and equipment cost. Therefore, in practical FOWLP technology, the maximum thickness of the dielectric layer achievable by the RDL process is limited to 15 µm. Reducing line width and spacing improves system integration, but it requires more sophisticated equipment and rigorous process control. To reduce process costs, the minimum line width of the RDL process is limited to 20 µm.

**Figure 2:**
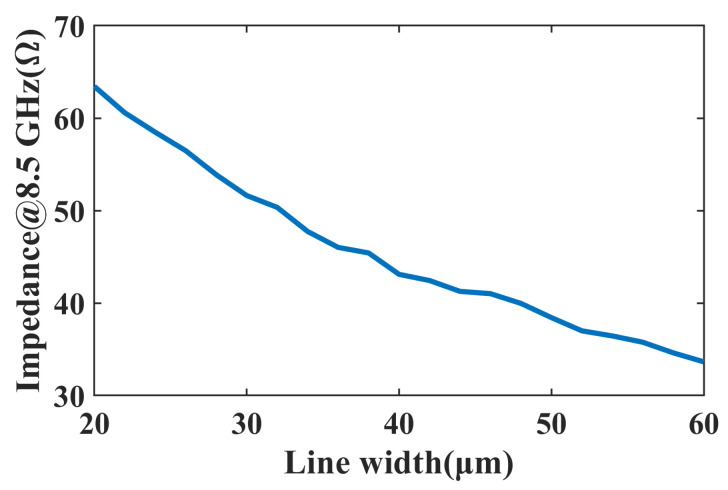
Impedance variation with microstrip line width on a 15 µm dielectric layer.

The impedance of the microstrip line versus the width of the line on a single dielectric layer of 15 µm thickness is shown in Figure 2. It can be seen that the minimum linewidth of the single dielectric layer corresponds to a maximum impedance of no more than 65 Ω. However, according to the design principles of the Wilkinson power divider, if the characteristic impedance is 50 Ω, the maximum impedance of the power divider should reach 70.7 Ω.

**Figure 3:**
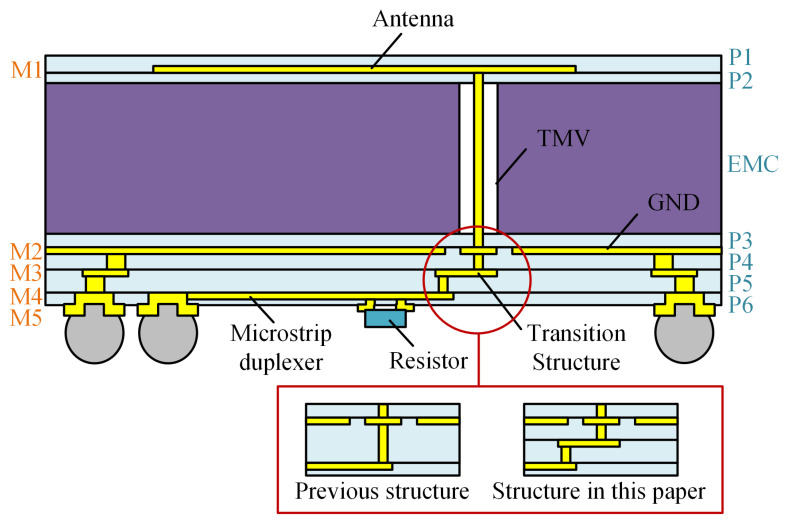
Stack-up of the IPD structure (where ‘Previous structure’ refers to [22]).

Obviously, the maximum impedance achievable by a single dielectric layer in the actual process conflicts with the demanded impedance of the Wilkinson power divider and cannot satisfy the impedance requirements of the designed structure. For this problem, we use 2-layer RDL to fabricate the structure from the ground layer to the power divider layer, and the thickness of each dielectric layer is 15 µm, to increase the maximum achievable impedance. In order to avoid the increased process difficulty and risk caused by multiple stacked vias, we designed a new IPD structure, shown in Figure 3, in which the TMV chip is embedded in the EMC substrate and used as a vertical feed for the antenna. The design utilizes the double-sided redistribution layer (RDL) technique to fabricate different passive structures on the top and bottom sides of the EMC, where P1–P6 are the dielectric layers and M1–M5 are the copper metal layers. Compared to the structure proposed in the literature [22], the new structure adds a microstrip transition structure located on the M3 layer, allowing for a smaller thickness of the single RDL dielectric layer, thus reducing the development cost and the fabrication difficulty of the FOWLP process.

In the structure of Figure 3, The upper redistribution structure comprises two dielectric layers and one copper layer, where M1 serves as the antenna. The lower redistribution structure includes four dielectric layers and four copper metal layers, each fulfilling a specific role: M2 is the ground plane; M3 provides a 50-ohm microstrip transition structure; M4 is a microstrip duplexer, which is a Wilkinson power divider for full-duplex transceiver of signals; M5 is the pad layer of solder balls and an isolation resistor, on which 500 µm-diameter solder balls are attached, as well as a 0201-sized resistor with dimensions of 0.6 × 0.3 × 0.23 ${\mathrm{mm}}^{3}$ and a 100 Ω resistance value. The materials and structural parameters used in the FOWLP process are provided in Table 1 [23,24].

### 2.2. Preparation Process of the IPD Structure

**Figure 4:**
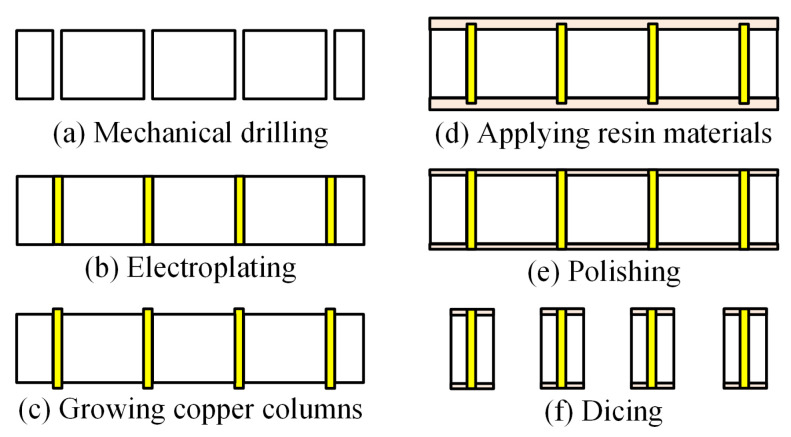
TMV process flow.

Before fabricating the structure, a 1 × 1 through-mold via (TMV) chip needs to be prepared as the vertical feed of the antenna. The key steps in the TMV fabrication process include drilling and through-hole metallization. The drilling technology includes laser drilling, mechanical drilling, and chemical etching; metallization is typically achieved through the electroplating process. Among various methods, the integration of mechanical drilling with electroplating is adopted due to its maturity, cost-effectiveness, and reliability in ensuring the quality of through-holes [23]. The fabrication process of the TMV chip is illustrated in Figure 4. First, mechanical drilling is performed on the core board to form through-holes. Then, electroplating is carried out to fill the through-holes with conductive materials such as copper. Next, copper columns are grown on both sides of the conductive through-holes, and resin materials are applied to protect the structure. The front and back sides of the core board are polished to remove excess material and expose the copper columns. Finally, dicing is performed to obtain the desired TMV array.

**Figure 5:**
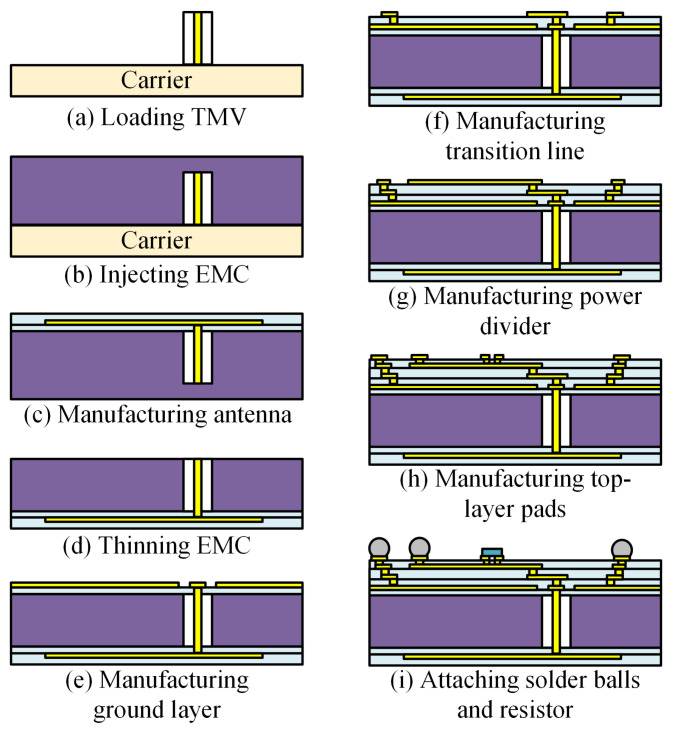
IPD structure process flow.

The detailed fabrication process of the FOWLP technology based on TMV chip is illustrated in Figure 5. First, the temporary carrier wafer is cleaned, and then a surface bonding film with good flatness and viscosity is attached to it to effectively adhere the TMV chip. Subsequently, the TMV chip is picked up by the pick-and-place machine based on the preset coordinates and placed on the temporary bonding substrate, as shown in Figure 5a. After that, the compression molding process is adopted, and epoxy molding compound (EMC) is injected to accomplish the wafer-level encapsulation of the TMV chip, as shown in Figure 5b. Then, the temporary carrier wafer is removed. The antenna structure is fabricated on the upper surface of the EMC layer using the redistribution layer (RDL) process. First, the upper surface of the EMC is thinned to expose the copper pillars of the TMV chip. Then, a 10-µm-thick layer of polyimide (PI) is deposited on the surface as the protective layer, involving processes such as coating, baking, exposure, and development. The PI layer is patterned with 40-µm-radius holes, smaller than the TMV copper pillar’s radius. Next, Ti is sputtered as an adhesion layer, followed by Cu sputtering as a seed layer. Copper electroplating is performed to build up the Cu layer to a thickness of 5 µm, thereby forming the antenna structure. Finally, an insulating dielectric layer is deposited on the Cu layer to protect the antenna structure in subsequent processing steps, as shown in Figure 5c. The wafer is flipped and undergoes backside thinning until the copper pillars of the TMV chip are exposed, as shown in Figure 5d. On the backside, the RDL process is performed, involving the alternating deposition of four PI layers and four copper layers. This process sequentially fabricates the ground plane, the transition line structure, the Wilkinson power divider, and pads for solder balls and surface-mounted resistors, as shown in Figure 5e–h. Finally, Sn-3.0Ag-0.5Cu (SAC305) solder bumps with a diameter of 500 µm and the 0201-sized resistor are surface-mounted using the reflow soldering process, as shown in Figure 5i.

### 2.3. Test Method of the IPD Structure Based on Vertically Soldered SMPM Interface

The traditional antenna testing method is to feed the antenna through the SubMiniature version A (SMA) connectors to measure S-parameters and radiation patterns. For AiP structures, a probe feed is typically used to evaluate these parameters [12]. However, neither of these methods is suitable for the structure presented in this paper. First, the SMA interface size is relatively large, with the need to match the design of larger pads; however, the substrate thickness on the back of the structure is too thin, with a thickness of only 30 µm between the pad and the ground layer. It is difficult to achieve impedance matching only through the microstrip pads, so there is a need to introduce additional resistance, capacitance, or inductance for impedance matching, thus increasing the complexity of the process. Second, most AiP antenna test platforms are typically equipped with only one test probe; however, the proposed IPD structure, which is applied to near-range radar detection, has two ports for transmitting and receiving signals, respectively. This requires that when one port is fed, the other port must maintain impedance matching and also needs to be able to test the antenna’s far-field radiation pattern, which makes it difficult for existing probe test methods to meet the test requirements of the IPD structure. Third, the IPD structure is originally designed as the top layer of an antenna and RF integrated system to be stacked and interconnected with subsequent structures via solder balls. The backside of the IPD structure has been optimized for the process requirements and impedance matching of coaxial-like structures; however, this spacing is not compatible with the dimensions of a standard ground-signal-ground (GSG) probe test interface. Conventional GSG probes have a spacing of 150 µm, whereas the solder ball pads in IPD structures have a spacing of at least 1 mm, which results in conventional probes not being able to be directly matched to the structures proposed in this paper for effective testing, increasing the difficulty and cost of testing.

**Figure 6:**
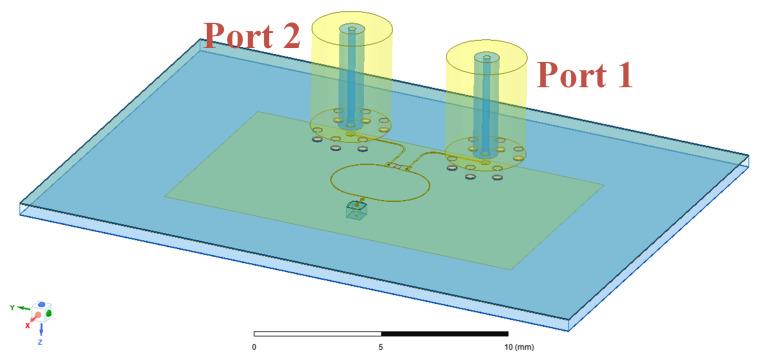
FEM simulation model.

To solve this problem, we designed a test method for vertical feed and created a finite element (FEM) simulation model, as shown in Figure 6. The IPD structure is connected to other substrates via solder balls, with the pitch between the center solder ball and the surrounding ones matching the dimensions of a SubMiniature Push-On Micro (SMPM) connector. Two SMPM connectors are then soldered to the back of the IPD structure. In Figure 6, Port 1 is used for feeding, simulating the input power from the transmitter of the FMCW system to the IPD structure. At the same time, Port 2 is connected to a 50-ohm load, simulating the receiver connection. This innovative approach of vertically soldering SMPM connectors not only addresses the testing challenges posed by the IPD structure’s unique design but also enables efficient measurement of return loss, isolation, and far-field radiation patterns. We also incorporate the solder ball model into the HFSS simulation to increase the simulation model’s accuracy and reliability, enabling it to better simulate the testing results.

### 2.4. Dimensions and Simulation Results of the Integrated Passive Device

**Figure 7:**
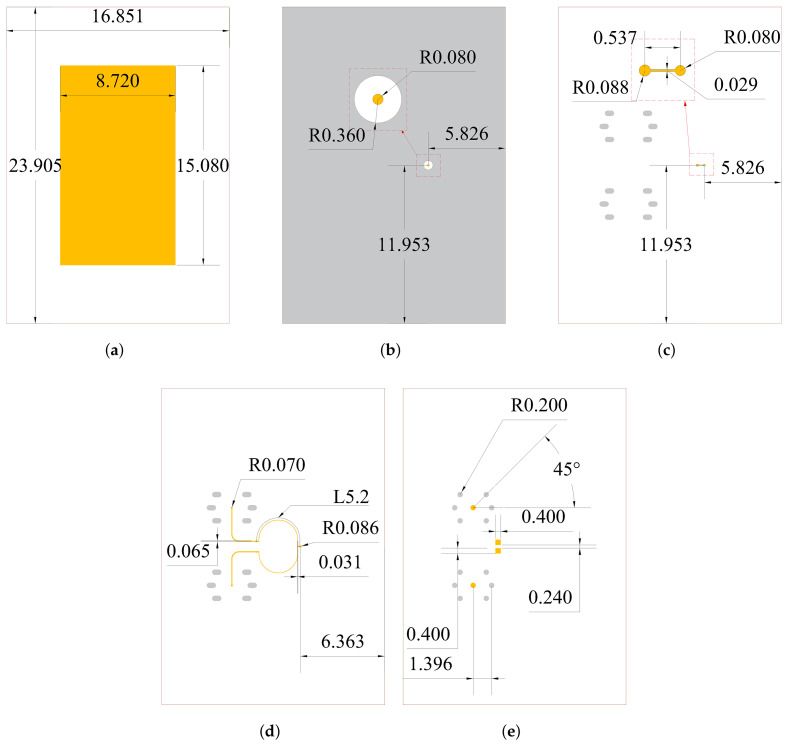
Schematic of (**a**) M1, (**b**) M2, (**c**) M3, (**d**) M4, (**e**) M5 of the proposed IPD (length unit: mm). The colored areas represent metal Cu: yellow corresponds to the RF signal, and gray corresponds to the ground.

**Figure 8:**
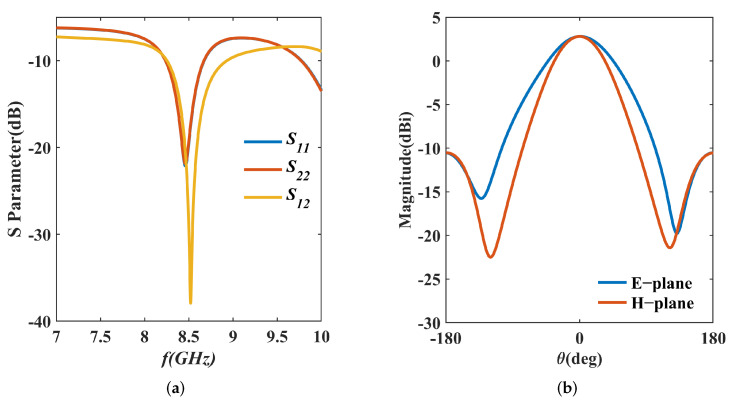
Simulation results of the proposed IPD structure: (**a**) S parameters; (**b**) E−plane and H−plane.

The geometry of the IPD structure is shown in Figure 7, and the structure is dimensioned with 8.5 GHz as the center frequency. To meet the minimum required spacing of 20 µm between the metal edges and the via holes, circular pads are introduced at connection points. The simulation results are illustrated in Figure 8. It shows that the return loss of the IPD structure is lower than −10 dB from 8.23 to 8.67 GHz, which means the working bandwidth of the structure is 5.21%. The maximum isolation within the working bandwidth exceeds 30 dB. The far-field radiation patterns, simulated with a center frequency of 8.5 GHz, indicate that the maximum gain is 2.80 dBi. The simulation results theoretically demonstrate the feasibility of the IPD structure with SMPM connectors.

Since the Wilkinson power divider is used as the duplexer, there is at least a 3 dB power loss, resulting in a relatively low antenna gain. To improve the system gain, an antenna array could be used to compensate for this loss. For an FMCW short-range radar system, with a transmitter power of 10 dBm, we can use the radar equation to compute the required gain for measuring distances within six meters. Based on the data in Table 2 and the radar equation [9], the IPD structure only needs to provide a gain of 1.57 dBi to theoretically measure distances up to 6 m. The proposed IPD structure satisfies this requirement. Optimizing the structure’s gain is beyond the scope of this paper and can be addressed in future work.

## 3. Measurement of the Integrated Passive Device Structure

**Figure 9:**
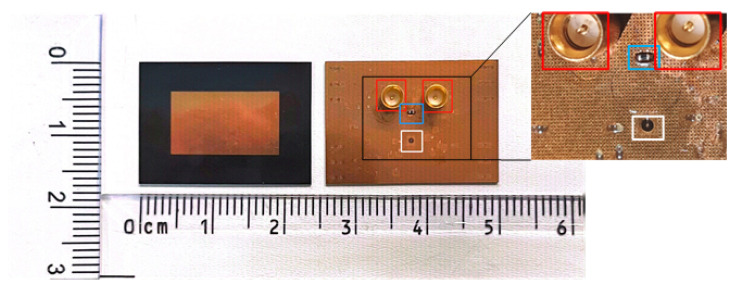
Photographs of the front and back of the proposed IPD structure. The red box indicates the SMPM connectors, the white box shows the TMV chip, and the blue box highlights the surface-mounted 0201-sized resistor.

**Figure 10:**
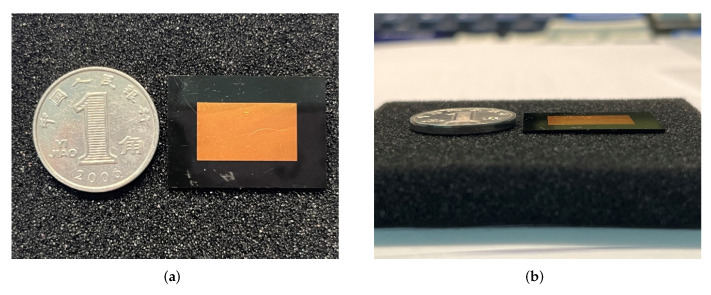
Size comparison of the IPD structure with a standard coin: (**a**) top view; (**b**) side view.

To further verify the feasibility of the designed structure, the proposed IPD structure is fabricated as shown in Figure 9. Figure 9 also displays the Wilkinson power divider on the backside of the IPD following SMPM soldering. This provides a clear view of the divider’s physical form, as well as its routing and the via holes in the ground layer. These via holes are intended to alleviate internal wafer stresses during the manufacturing process. Subsequent measurements showed that these via holes had no adverse effects on the IPD system’s functionality. Figure 10 presents the size comparison between the fabricated IPD structure and a standard coin, highlighting the IPD’s thin profile and the precision of our fabrication process. The total dimension of the structure’s substrate is 16.983 × 24.099 × 0.56 ${\mathrm{mm}}^{3}$.

**Figure 11:**
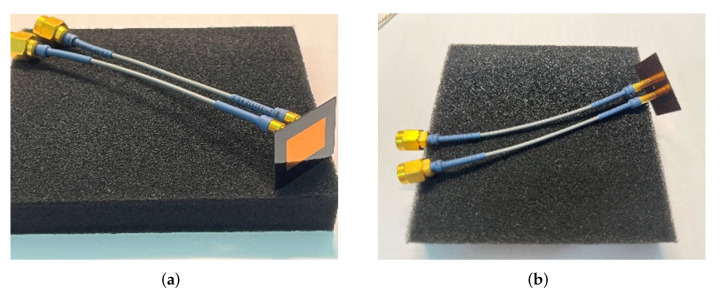
Schematic of cable connections to the IPD structure: (**a**) front view; (**b**) back view.

**Figure 12:**
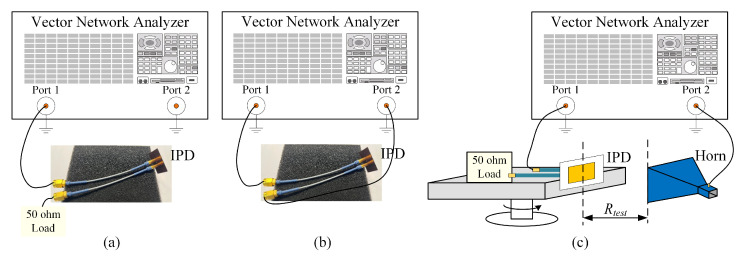
Test configuration schematics of the IPD structure: (**a**) $S_{11}$; (**b**) $S_{12}$ (isolation); (**c**) far-field radiation pattern.

As shown in Figure 11, two 10 cm long SMPM-to-SMA coaxial cables are connected to both ends of the IPD structure. Prior to measurements, the vector network analyzer (VNA, E8363B, Keysight Technologies, Santa Rosa, CA, USA) is configured for the 7–10 GHz frequency range with a source power of 4 dBm. One-port calibration is implemented utilizing calibration standard connectors (open, short, load). As shown in Figure 12a, the cable connected to Port 1 of the IPD structure is linked to Port 1 of the vector network analyzer, while the cable connected to Port 2 of the IPD structure is attached to a 50-ohm matching load for measuring the return loss $S_{11}$. Then, we used a through calibration standard connector to connect Port 1 and Port 2 of the VNA to perform through calibration. Next, we connected the IPD structure to the VNA, as shown in Figure 12b, and measured $S_{12}$, which is equivalent to the isolation of the IPD structure. As shown in Figure 12c, for measuring the far-field radiation patterns of the IPD structure, an X-band horn antenna is connected to Port 2 of the VNA and used as the receiver. Port 1 of the IPD structure remains connected to Port 1 of the VNA, and the IPD structure is placed on a rotating platform with a rotation range of −180° to 180°. The rotating platform and the horn antenna are positioned on the same plane. The quantity $R_{test}$ is the horizontal distance between the IPD structure and the horn antenna, which is 2 m in the experiments conducted in this paper. This distance value must be greater than the far-field distance of the antenna. For measuring antenna gain, we replaced the IPD structure in Figure 12c with a horn antenna of known gain, ensuring that the center of the horn antenna aligns with the spatial coordinates of the IPD structure. We recorded the $S_{12}$ value measured by the VNA as the reference value. Then, the IPD structure was replaced again to measure the $S_{12}$ value. The gain of the IPD structure was calculated based on the $S_{12}$ value corresponding to the standard horn antenna and the gain of the horn antenna.

**Figure 13:**
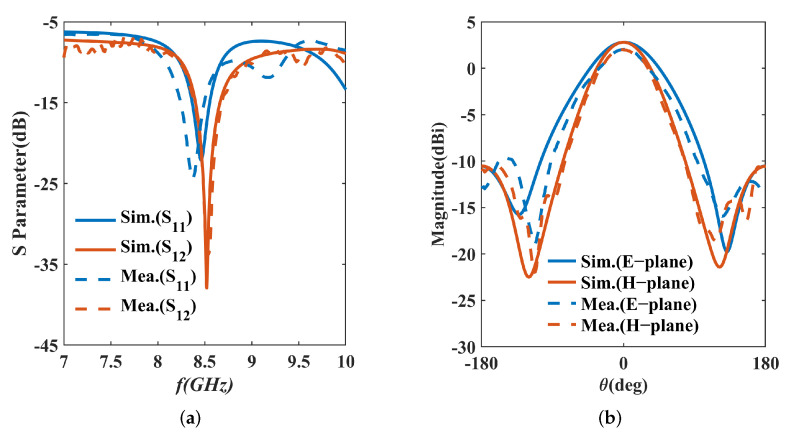
Simulation and measured results of the proposed IPD structure: (**a**) S parameters; (**b**) E−plane and H−plane.

The corresponding results are presented in Figure 13. For return loss, the measured $S_{11}$ shows a minimum at 8.365 GHz with −24.38 dB, while the simulated $S_{11}$ is at 8.46 GHz with −22.11 dB. There is a resonance frequency shift. At the center frequency of 8.5 GHz, the measured $S_{11}$ value is −14.62 dB, which is higher than the simulated value of −19.42 dB. The measured bandwidth is 7.66% (8.095–8.74 GHz), up from the simulated 440 MHz to 645 MHz. For maximum isolation, the measured value is 33.88 dB at 8.53 GHz, compared to the simulated 37.93 dB at 8.52 GHz. The transceiver isolation ($S_{12}$) of the FMCW system cannot be less than 20 dB [25], and the measured bandwidth with isolation higher than 20 dB is 1.76%, ranging from 8.455 GHz to 8.605 GHz, which is slightly wider than the simulated bandwidth of 1.58% (8.456–8.59 GHz). The measured far-field radiation pattern at 8.5 GHz shows a maximum gain of 2.02 dBi, lower than the simulated 2.8 dBi. Despite this, the gain still meets the FMCW system’s needs for short-range applications. The comparison between the simulated values and test values of the main parameters is shown in Table 3.

**Figure 14:**
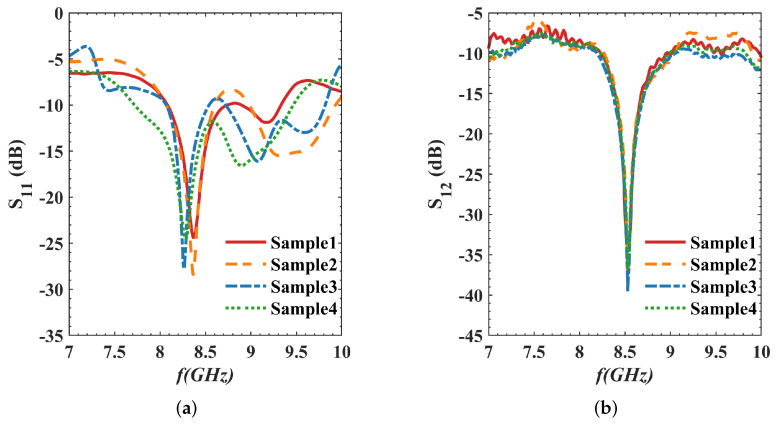
S parameters of different IPD structures: (**a**) return loss $S_{11}$; (**b**) isolation $S_{12}$.

**Figure 15:**
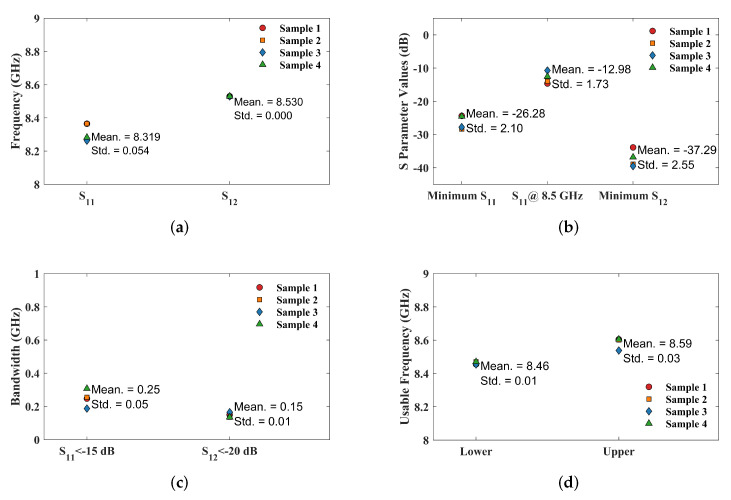
Parameter distributions of different IPD sample structures: (**a**) frequencies corresponding to the minimum S parameter values; (**b**) S parameter values; (**c**) bandwidths; (**d**) upper and lower limits of the usable frequency range.

Figure 14 shows the $S_{11}$ and $S_{12}$ curves for four IPD sample structures. The figure shows that there are some differences between the $S_{11}$ curves of each sample, while the $S_{12}$ curves are basically consistent. Figure 15 illustrates the uncertainty analysis of parameters from these samples. As shown in Figure 15a,b, for $S_{11}$, the resonant frequency averages 8.319 GHz (with a standard deviation of 0.054 GHz), the minimum $S_{11}$ value averages −26.28 dB (with a standard deviation of 2.1 dB), and, at the center frequency of 8.5 GHz, $S_{11}$ averages −12.98 dB (with a standard deviation of 1.73 dB). The lowest points of $S_{12}$ for the four samples all correspond to a frequency of 8.53 GHz, demonstrating good consistency. The minimum $S_{12}$ value (linked to maximum IPD isolation) averages −37.29 dB (with a standard deviation of 2.55 dB). For bandwidth, if $S_{11}$ < −10 dB is used as the criterion, the bandwidth in Figure 14a can reach over 1 GHz. To more comprehensively evaluate the consistency of the IPD structure, we use the bandwidth range when $S_{11}$ is below −15 dB for comparative analysis. As shown in Figure 15c, the average bandwidth for $S_{11}$ < −15 dB is 0.25 GHz, with a standard deviation of 0.05 GHz; and the average bandwidth for $S_{12}$ < −20 dB is 0.15 GHz, with a standard deviation of 0.01 GHz. For the latter two ($S_{11}$ < −15 dB and $S_{12}$ < −20 dB), the results exhibit a high degree of consistency. The intersection of the bandwidth ranges of $S_{11}$ < −10 dB and $S_{12}$ < −20 dB is taken as the usable frequency band for the IPD structure, as shown in Figure 15d. The lower limits of the usable frequency bands for the four IPD structures are concentrated around 8.46 GHz, and the upper limits are concentrated around 8.59 GHz, with a relatively uniform distribution. Although all IPD structures are derived from the same batch of wafer fabrication and utilize the same process flow and parameters, minor process variations may still occur during manufacturing, which have a greater impact on $S_{11}$ than on $S_{12}$.

**Figure 16:**
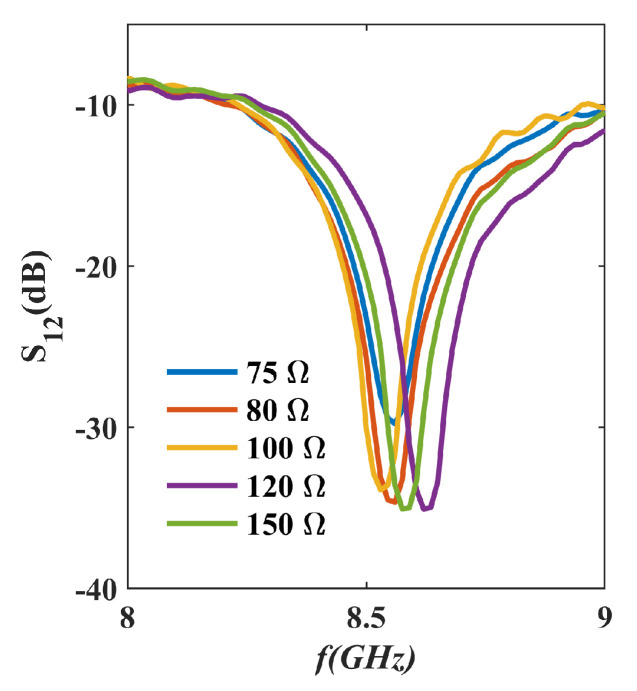
The S_12_ plot of the IPD structure with isolation resistors of different values.

Considering the principle of isolation for the Wilkinson power divider [26], the resistance value $R_{0}$ and its distance $L_{R0}$ relative to the 70.7 Ω microstrip line significantly influence the isolation performance. Given that the length of $L_{R0}$ becomes fixed once the IPD structure is fabricated and is not easily altered, we adjust the resistance value $R_{0}$ and conduct isolation tests, as shown in Figure 16. The results demonstrate that, at the central frequency of 8.5 GHz, the optimal isolation performance of the power divider is achieved with a resistance of 100 Ω, achieving an isolation value of 30.06 dB. However, within the 8–9 GHz frequency band, the isolation performance peaks at a resistance of 120 Ω, yielding an isolation value of 35.07 dB at 8.62 GHz.

To highlight the advantages of the proposed IPD structure, a comparison with other FMCW AiP technologies is presented in Table 4, which shows that the IPD structure features a thin substrate (0.016 $\lambda_{0}$), multi-functionality (integrating an antenna and a microstrip duplexer), and compatibility with high-volume fabrication and integration (FOWLP process and solder ball stacking). These advantages promote the application of the FOWLP process in the integration of passive components for X-band systems, and more kinds of passive components can be integrated using FOWLP in the future.

## 4. Conclusions

This paper focuses on the fabrication and testing of the IPD structure for passive microwave components in the X-band FMCW radar using the FOWLP process. First, the dielectric layer in the RDL process of FOWLP is utilized as the substrate for microwave components, and the impedance upper limit value is improved by adding a transition layer structure to achieve low-cost and low-difficulty FOWLP process fabrication of the IPD structures; second, the testing difficulty of the dual ports for the double-sided microwave structure with an antenna is solved by adopting a vertically soldered SMPM interface, and the full-wave electromagnetic field of the structure with the test interface is simulated. Third, the FOWLP process fabrication and testing of the IPD structure are completed, and the test and simulation results are similar, verifying the performance of the structure in an X-band FMCW radar detection system. Additionally, experimental uncertainty analysis is conducted. Based on the distribution diagram of the samples, the test results of different IPD structures are generally consistent, verifying the stability of the design and providing a new solution for system miniaturization.

## Figures and Tables

**Table 1:** Structural and material parameters of the FOWLP process used in this paper.

Name	Length (µm)	Width (µm)	Thickness (µm)	Radius (µm)	Permittivity	Tan(δ)	Material
M1–M5	**—**	**—**	5	**—**	**—**	**—**	Cu
P1 P2, P3, P6 P4, P5	**—**	**—**	25 10 15	**—**	3.3	0.008	RDL dielectric
TMV dielectric	600	600	450	**—**	4.4	0.01	Core
TMV via	**—**	**—**	450	60	**—**	**—**	Cu
EMC	**—**	**—**	450	**—**	3.69	0.006	EMC

**Table 2:** Descriptions and values of FMCW ranging system parameters.

Characters	Descriptions	Values
$R_{\max}$	Maximum measurement distance	6 m
$\delta_{R}$	Receiver sensitivity	−80 dBm
$P_{t}$	Transmitter power	10 dBm
λ	Wavelength	0.0353 m
$\sigma_{T}$	Target cross-section	1 m^2^

**Table 3:** Comparison of simulated and measured IPD structure performance.

Parameters	Simulated Values	Measured Values
Resonance frequency	8.460 GHz	8.365 GHz
Minimum $S_{11}$	−22.11 dB	−24.38 dB
$S_{11}$ @ 8.5 GHz	−19.42 dB	−14.62 dB
Bandwidth ($S_{11}$ < −10 dB)	440 MHz	645 MHz
Maximum isolation	37.93 dB @ 8.52 GHz	33.88 dB @ 8.53 GHz
Bandwidth (iso. > 20 dB)	134 MHz	150 MHz
Peak gain @ 8.5 GHz	2.80 dBi	2.02 dBi

**Table 4:** Comparison of this work and other FMCW AiP technologies.

Work	Technique	Frequency (GHz)	Size ($\lambda_{0}^{3}$)	Integration Level of Passive Devices	Interconnection Method to MMIC	Relative Position to MMIC
[13]	Silicon	88–96	9.09 × 4.99 × 0.156	1 Tx/2 Rx antenna array	RDL	Horizontal
[14]	LTCC	122	2.85 × 2.40 × 0.171	1 Tx/1 Rx antenna array	Wire bonding	Horizontal
[15]	LTCC	8–12	0.38 × 0.65 × 0.095	1 full-duplex Tx/Rx antenna array	Wire bonding	Vertical
[17]	HDI	94	3.76 × 3.76× 0.155	1 Tx/1 Rx antenna array	Flip-chip	Vertical
[18]	PCB	60	More than 0.34 × 0.268 × 0.06	1 Tx antenna	Wire bonding	Horizontal
[19]	FOWLP	61 122	1.63 × 1.63 × 0.071 3.25 × 3.25 × 0.142	1 Tx/1 Rx antenna array	RDL	Horizontal
This work	FOWLP	8.5	0.48 × 0.68 × 0.016	1 full-duplex Tx/Rx antenna and a microstrip duplexer	RDL, solder ball stacking	Vertical

## Data Availability

The original contributions presented in this study are included in the article. Further inquiries can be directed to the corresponding author.
